# *Nitzschiatranstagensis* Morales, Novais, Wetzel, Morais & Ector (Bacillariophyceae) – the second record in the Mediterranean Region

**DOI:** 10.3897/BDJ.12.e115601

**Published:** 2024-03-19

**Authors:** María Cid-Rodríguez, Lorena González-Paz, Cristina Delgado

**Affiliations:** 1 University of Vigo, Vigo, Spain University of Vigo Vigo Spain

**Keywords:** diatom, distribution, ecology, epilithon, taxonomy

## Abstract

**Background:**

The Mediterranean Region holds significant ecological importance, characterised by its unique climate, biodiversity and the crucial role it plays in global ecosystems. Mediterranean streams are naturally highly-stressed environments mainly due to fluctuations in water quantity. River flow generally varies from perennial to ephemeral and temporary rivers constitute significant water resources. Streams that flow through Balearic Islands are subjected to these conditions. The majority of these streams sustain water flow for 4–5 months annually, with exceptions noted for streams associated with springs, which typically maintain water throughout most of the year.

Benthic diatoms are widely recognised as reliable bioindicators of water quality, used in many aquatic ecosystems. Analysing diatom communities and their biodiversity serves as a valuable tool to ensure the ecological and sustainable utilisation of water resources as well as the accurate development of guidelines for their conservation.The field of diatom taxonomy and distribution plays a crucial role in advancing our understanding of aquatic ecosystems and their biodiversity. Species of the genus *Nitzschia* are extensively found throughout the Mediterranean Region, including the Balearic Islands. However, they have rarely been investigated in temporary streams.

**New information:**

This study presents the first record of *Nitzschiatranstagensis* Morales, Novais, Wetzel, Morais & Ector, outside the type locality and being the second record in Europe. In this study, the authors found this taxon in one temporary stream of Majorca Island, Torrent des Castellot in November 2005 (Balearic Islands). *Nitzschiatranstagensis* occurred at 2.6% abundance in this stream with oligotrophic waters (0.052 mg∙l^-1^ of nitrate), slightly alkaline pH values (7.8) and water conductivity levels of 626.5 µS cm^-1^. This species was recorded in the biofilm of the stones together with other taxa such as *Achnanthidiumminutissimum* (Kützing) Czarnecki (39.2%), *Gomphonemarosenstockianum* Lange-Bertalot & Reichardt (28.9%) and *Halamphoraoligotraphenta* (Lange-Bertalot) Levkov (20.4%). The *Nitzschiatranstagensis* frustules found in the examined material have similar dimensions and a higher fibulae count (8–11 in 10 μm vs. 6–10 in 10 μm) compared to the type material of *Nitzschiatranstagensis*. The habitat characteristics in which this species was found are described, together with LM micrographs of this taxon.

## Introduction

Species belonging to the genus *Nitzschia* Hassal are widely distributed in the Mediterranean Region, including the Balearic Islands. *Nitzschia* consists of 895 taxonomically-accepted species (Guiry and Guiry 2023), which have been found in a diverse range of habitats, both in the benthos and the plankton of freshwater, brackish and marine environments (Mucko et al. 2021). *Nitzschia* genus was first described in 1845 by Hassall, unifying all single-celled and colonial pennate diatoms with a linear to lanceolate (also sigmoid) shape of frustules and with a predominantly eccentric (sometimes centric) raphe subtended by siliceous bridges (Giulietti et al. 2021). Smith (1853) divided this large genus into six sections for the first time. Later, Cleve and Grunow (1880) further subdivided the genus *Nitzschia* into 24 sections, based mainly on the shape of the valves, organisation and structure of the fibulae and raphe position (Krammer and Lange-Bertalot 1988). Grunow’s system is still in use with some modifications, particularly following Mann (1986) and Round et al. (1990). Mann (1986) reviewed the classification of *Nitzschia* and set up the subgenusNitzschia, which is characterised by a complex structure of the valve near the raphe, with the presence of siliceous flaps, an undulate valve face and changes in valve structure beneath the conopeal canals. This unique combination, known as the canal-raphe-conopeum system, is not found in other groups of *Nitzschia* species. The *Lineares* group was first described by Grunow (1862), whereas Cleve (1883) was the first to assign sectional status to this group. Morales et al. (2019) described a new *Nitzschia* species in the Linearessectionsensu Krammer and Lange-Bertalot (1988). The first record of *Nitzschiatranstagensis* Morales, Novais, Wetzel, Morais & Ector occurred in a spring feeding the small creek Barranco do João Dias in the Portuguese region of Alentejo (Morales et al. 2019). Therefore, this study aims to increase the knowledge about the *N.transtagensis* habitat by describing the ecological data of a new locality and to provide light microscopy documentation of this species.

## Materials and methods

### Study area

**Figure 1.**
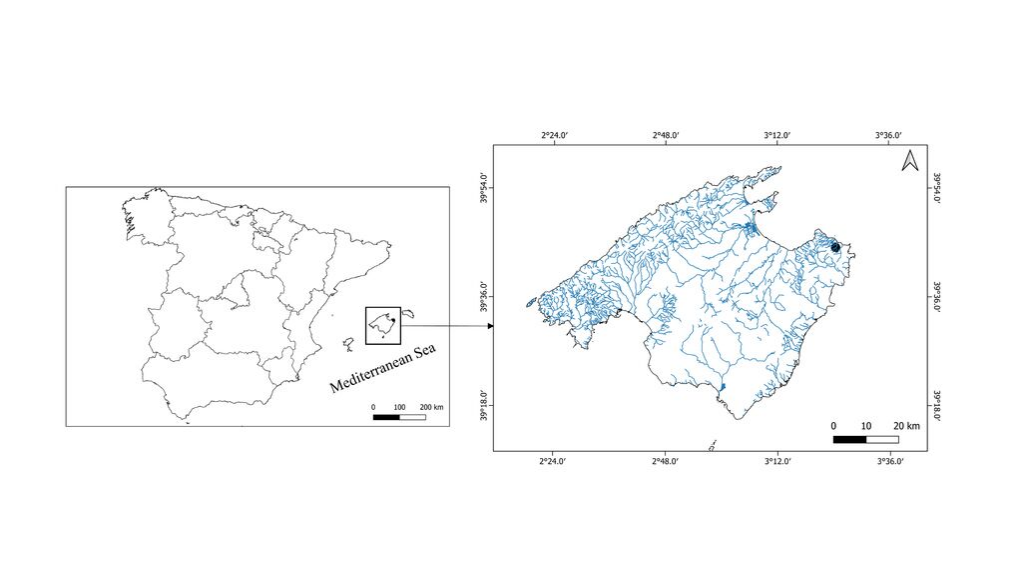
Map of the island of Majorca showing the location of Torrent des Castellot (black circle). The left side shows its location within Spain.

The Balearic Islands (Fig. 1) possess a hydrological system comprising of temporary streams which are strongly influenced by the Mediterranean climate. These streams result from predictable and seasonal flooding and drying events during the annual hydrologic cycle. They tend to sustain a stable community between September-October towards April-May, after which they experience a prolonged period of approximately 4 months of drying. Information on land-use cover in the areas near the stream was estimated from CORINE land-cover maps (Bossard et al. 2000). The vast majority was dedicated to forested regions (70%) and agricultural activities (15%) and, to a lesser extent, to natural vegetation (15%).

This study is part of a larger project conducted during 2005-2006 which involved sampling 60 temporary streams located in the Balearic Islands (Spain) during different seasons (winter, spring and autumn) (Delgado et al. 2012).

### Diatom samples

Water temperature (ºC), pH, dissolved oxygen (mg l^-1^) and electric conductivity (EC, µS cm^-1^) were measured *in situ* using portable meters. Water chemical analyses followed the American Public Health Association methods (APHA 1989). BOD_5_ was measured using the oxitop WTW; alkalinity by the potentiometric method. For nutrient analysis, an Auto-Analyzer 3 (Bran + Luebbe, Germany) was used. Ions were measured using a spectrophotometer of masses, whereas chlorides (Cl-) and sulphates (SO_4_^2-^) were quantified using Inductively Coupled Plasma-Mass Spectrophotometry (ICP-MS) (further information in Delgado et al. (2012)).

Epilithic diatoms were collected from stones using a small toothbrush, following European protocols (Kelly et al. 1998, AFNOR 2003, CEN - Comité Europeén de Normalisation 2004). After collection, diatom samples were preserved with a formaldehyde solution (37%). Subsequently, they were digested using the procedure of Renberg (1990) with hydrogen peroxide (H_2_O_2_) and, following oxidation, permanent slides were prepared with Naphrax®. A minimum number of 400 valves were identified and counted from each slide under a Nikon Eclipse E800 light microscope (LM), equipped with an immersion objective 100x (NA 1.40) to assess the relative abundance of taxa. Light micrographs were captured using a DS-U2 digital camera and NIS-Elements D 2.30 SP1 software (Nikon, Japan). The diatoms were identified at the lowest taxonomical level according to reference floras: Krammer and Lange-Bertalot (1991), Lange-Bertalot (1993), Krammer (1997a), Krammer (1997b), Lange-Bertalot et al. (2003), Hofmann et al. (2011), Lange-Bertalot et al. (2017).

To assess the ecological status, the most common diatom indices were calculated using the Omnidia software v. 5.3 (Lecointe et al. 2003): the Specific Polluosensitivity Index (IPS, CEMAGREF and AERMC (1982)), the Biological Diatom Index (IBD, Prygiel and Coste (1999)), the Trophic Diatom Index (TDI, Kelly and Whitton (1995)) the Commission for Economical Community Metric (CEE, Descy and Coste 1991), the Indice Diatomique Artois Pircardie (IDAP, Prygiel et al. (1996), Lecointe et al. (2003)) and the Shannon–Wiener Diversity Index (*H*’, *Shannon and Weaver (1949)*).

## Taxon treatments

### Nitzschia transtagensis

E.Morales, Novais, C.E.Wetzel, Morais & L.Ector, 2019

68130BD7-FAEF-5F8A-A1C3-79E2A55CE623

#### Materials

- **Type status:** Other material. **Occurrence:** occurrenceDetails: https://www.tandfonline.com/doi/abs/10.1080/23818107.2019.1688676; recordedBy: Cristina Delgado; individualCount: 2.6%; associatedReferences: https://www.tandfonline.com/doi/abs/10.1080/23818107.2019.1688676; occurrenceID: F74F768A-9684-5D9C-A211-BFEA371756D9; **Location:** continent: Europe; waterBody: Temporay streams from Balearic Isands; islandGroup: Balearic Islands; island: Majorca; country: Spain; countryCode: ES; stateProvince: Majorca; county: Spain; municipality: Artà; **Event:** samplingProtocol: Light microscope count; year: 2005; habitat: Temporary stream

#### Description

**Figure 2.**
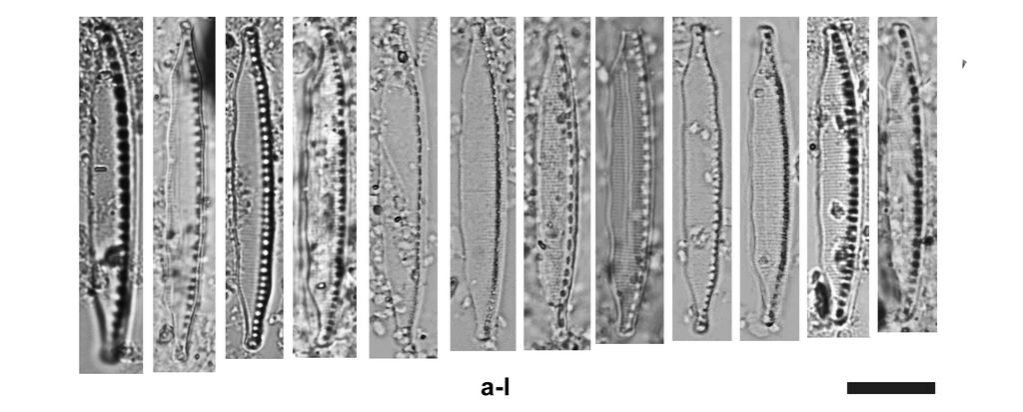
Light micrographs of *Nitzschiatranstagentis* Morales, Novais, Wetzel, Morais & Ector, found in a torrent of Castellot (Majorca Island). Scale bar = 10 μm.

Frustules with apical asymmetry with an undulated pattern on the secondary side (Fig. 2). Valves 34.6–40.0 μm long, 4.0–5.3 μm wide and fibula density 8–11/10 μm (n = 13). It is noteworthy that striae are visible with LM in some specimens. Directing attention to the images in Figure 2, we highlight the species distinctive characteristics, including a convex primary (raphe) side with a slight undulated abvalvar edge of the mantle and clearly undulated secondary valve side. We believe the debris surrounding the Nitzschia specimens may be attributed to the calcareous geology of Majorca. In the studied samples, *Nitzschiatranstagensis* frustules were observed with length and width measurements consistent to those of the type material described by Morales et al. (2019), but with a higher fibulae density 8–11 in 10 µm compared to 6–10 in 10 µm reported by Morales et al. (2019).

#### Distribution

*Nitzschiatranstagensis* was found in a single sample collected from the Torrent des Castellot Mountain stream located in the Artá Municipality, northeast of the Majorca Island (Balearic Islands), coordinates 39° 44' 16.24” N, 3° 24' 30.07” W (Fig. 1). This stream was sampled during the autumn of 2005 and spring of 2006. The Torrent des Castellot Mountain stream is a tributary of the Torrent de sa Font des Pí, which flows into Cala Torta, NE of Majorca Island. Notably, *Nitzschiatranstagensis* only occurred in one of the two samples collected from the Torrent des Castellot Mountain stream, accounting for an abundance of 2.6%. *Nitzschiatranstagensis* Morales, Novais, Wetzel, Morais & Ector was exclusively found in the sample collected in autumn of 2005 from this specific locality. We visited the same location in May 2006 and in March and May 2008 to determine if the taxon was there, but it was impossible to take samples because the stream was dry.

#### Ecology

**Figure 3.**
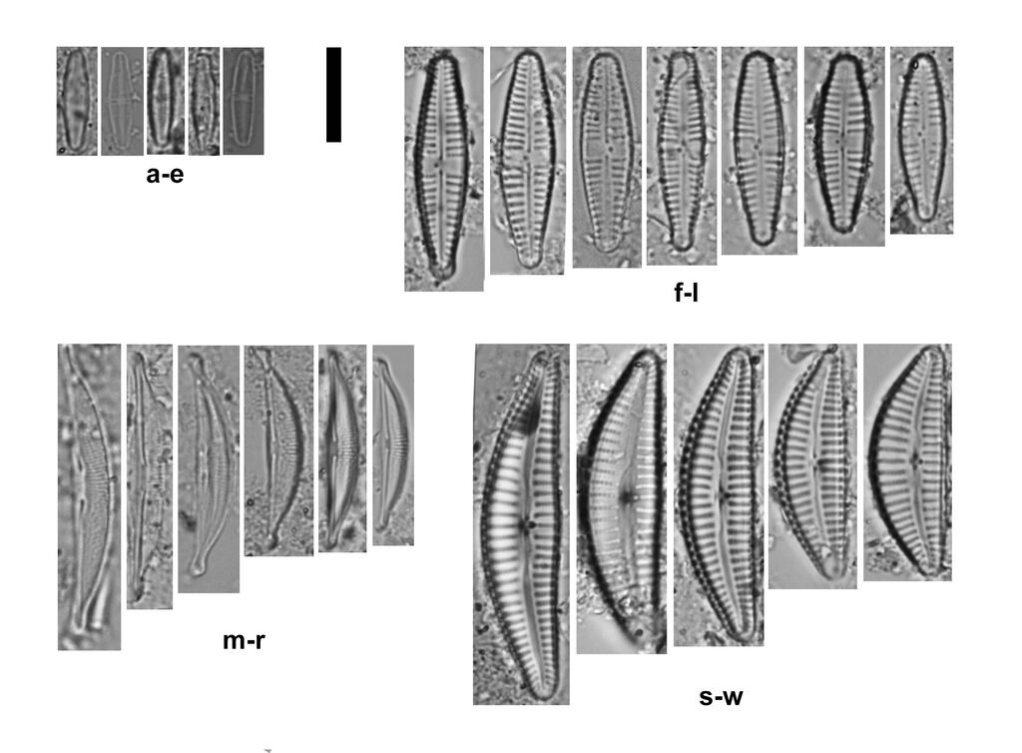
Light micrographs. **a-e** *Achnanthidiumminutissimum* (Kützing) Czarnecki; **f-l** *Gomphonemarosenstockianum* Lange-Bertalot & Reichardt; **m-r** *Halamphoraoligotraphenta* (Lange-Bertalot) Levkov; **s-w** *Cymbellavulgata* Krammer. Scale bar = 10 μm.

*Nitzschiatranstagensis* was present in oligotrophic waters (0.052 mg∙l^-1^ of nitrate), with slightly alkaline pH values (7.8) and medium water conductivity (626.5 µS cm^-1^). The values of these chemical parameters measured in May 2006 are provided in Table 1. The IPS index was 18.7 (over a maximum of 20) and the value of Shannon-Wiener (*H*’) diversity index was 2.26 (Table 2). Within the diatom assemblage where *N.transtagensis* was found, a total of 20 different diatom species were identified. These species were: *Achnanthidiumminutissimum* (Kützing) Czarnecki (39.2%) (Fig. 3a-e), *Gomphonemarosenstockianum* Lange-Bertalot & Reichardt (28.9%) (Fig. 3f-l), *Halamphoraoligotraphenta* (Lange-Bertalot) Levkov (20.4%) (Fig. 3m-r), *Naviculacryptotenella* Lange-Bertalot (2.3%), *Navicula* sp. 2 (1.6%), *Naviculacincta* (Ehrenberg) Ralfs (0.7%), *Craticulahalophila* (Grunow) D.G.Mann (0.5%), *Diploneisoblongella* (Nägeli ex Kützing) A. Cleve (0.5%), *Halamphoramontana* (Krasske) Levkov (0.5%), *Nitzschiafrustulum* (Kützing) Grunow (0.5%), *Naviculaveneta* Kützing (0.5%), *Encyonopsismicrocephala* (Grunow) Krammer (0.2%), *Cymbellavulgata* Krammer (0.2%) (Fig. 3s-w), *Encyonopsiscesatii* (Rabenhorst) Krammer (0.2%), *Fragilariarumpens* (Kützing) G.W.F.Carlson (0.2%), *Gomphonema* sp. Ehrenberg (0.2%), *Luticolagoeppertiana* (Bleisch) D.G.Mann ex Rarick, S.Wu, S.S.Lee & Edlund (0.2%), *Navicula* sp. (0.2%) and *Navicula* genus Bory (0.2%). In the sample collected in the spring of 2006, where *N.transtagensis* was not present, water temperature and electrical conductivity were higher (14.3ºC and 938.9 µS cm^-1^) and the assemblage was less diverse, being mainly dominated by *A.minutissimum* (92.3%) and *H.oligotraphenta* (3.8%).

## Discussion

In this study, we reported a new record of *Nitzschiatranstagensis* in a temporary stream on Majorca Island. Specifically, this species was found in the periphyton community attached to the submerged stones in the stream known as Tte des Castellot (Majorca). The habitat of *Nitzschiatranstagensis* was characterised by waters with medium electrolyte content, compared with water of other temporary streams of Majorca Island (Delgado et al. 2012), although somewhat higher than in the type locality from Portugal (626.5 vs. 237 µS cm^-1^) (Morales et al. 2019). The pH value at this temporary stream was slightly alkaline (7.8), whereas at the type locality, it was alkaline (8.4). Furthermore, the water temperature (7.8 vs. 21.3ºC) and the nutrient content (0.05 vs. 0.89 mg NO_3_ l^-1^; < 0.001 vs. 0.4 mg PO_4_ l^-1^) measured within this study were lower than those from the type locality spring. The study area demonstrated lower nutrient content compared to other calcareous geographical regions (Poikane et al. 2019). In regions characterised by higher nutrient content, nutrients emerged as the most influential variables in explaining the variation in diatom species composition (González-Paz et al. 2020). The genus *Nitzschia* is considered tolerant to pollution (Hill et al. 2001). In fact, it has been shown that an improvement in diatomological indices and, consequently, water quality, leads to a reduced occurrence of *Nitzschia* species (González-Paz et al. 2022). However, the occurrence of *Nitzschiatranstagensis* is more than four times higher in our locality, which has a low concentration of nutrients.

The most abundant species where *N.transtagensis* was found were *A.minutissimum*, *G.rosenstockianum* and *Halamphoraoligotraphenta*. These taxa are common in temporary streams across the Balearic Islands (Delgado et al. 2012, Delgado and Pardo 2015). All three species exhibit high IPS sensitivity values above 5.0. *A.minutissimum* has been reported from alkaline and acidic, oligotrophic and hypertrophic waters, being considered a ubiquitous species (Potapova and Hamilton 2007). Similarly, *G.rosenstockianum* has been found in oligo- to β-mesosaprobic waters, although it can be found in alpha-mesosaphrobic waters. It is considered an alcaliphilous species (Lange-Bertalot 1993, Novais et al. 2009). However, *Halamphoraoligotraphenta* is considered a reliable indicator for oligotrophic to weakly mesotrophic waters with low to average electrolyte content (Lange-Bertalot and Metzeltin 1996). The Mediterranean Region is already known as a biodiversity hotspot; furthermore, intermittent rivers are also well recognised as highly diverse (Novais et al. 2020). The Shannon-Wiener diversity value was high, similar to values reported in other Mediterranean temporary streams (Morais et al. 2004, Novais et al. 2020). Moreover, other studies state that IPS values above 17 suggest a high ecological status (Noga et al. 2014).

Morphological characteristics of our specimen conform to *Nitzschiatranstagensis* current description. Frustules found in the examined material have similar dimensions to those of the type material. The length and width of the frustules found in Majorca Island might be included within the dimensions proposed by Morales et al. (2019) in Portugal (34.6 – 40.0 μm long, 4.0 – 5.3 μm wide). However, in the study material, the fibulae range is higher (8–11 in 10 μm vs. 6–10 in 10 μm) compared to the type material (Morales et al. 2019). From a comparative examination of the description, our taxon corresponds to that described by Morales et al. (2019) and not to the Sardinian morphotype *Nitzschia* (aff.) *ebroicensis* reported by Lange-Bertalot et al. (2003) from Sardinian pools.

In literature, we did not find many other species of *Nitzschia* that could be confused with *Nitzschiatranstagensis*, frustules with nitzschioid symmetry, lanceolate with one convex side of the raphe and the opposite side distinctly undulated nor are common in the genus *Nitzschia*. For this reason, there is still a lack of information about the ecology of this species. Due to the presence only in one sample of Tte de Castellot (Majorca), the ecological preferences of *N.transtagensis* are still open.

## Supplementary Material

XML Treatment for Nitzschia transtagensis

## Figures and Tables

**Table 1.** Chemical parameters of the water stream where *Nitzschiatranstagensis* was found.

**Variable (autumn 2005)**	**Value**
pH	7.8
Electrical conductivity (µS cm^-1^)	626.5
Water temperature (ºC)	7.8
Dissolved oxygen (mg l^-1^)	10.7
Oxygen saturation (%)	89.5
Cl- (mg l^-1^)	42.5
SO_4_^2-^ (mg l^-1^)	30.8
Mg^2+^ (mg l^-1^)	49.9
S^2-^ (mg l^-1^)	1.2
Ca^2+^ (mg l^-1^)	47.4
Na^+^ (mg l^-1^)	64.9
K^+^ (mg l^-1^)	2.8
DBO (mg l^-1^)	< 2.0
SiO_2_ (mg l^-1^)	2.4
Fe^2+^ (mg l^-1^)	0.001
PO_4_^3-^ (mg l^-1^)	< 0.001
NO_2_- (mg l^-1^)	< 0.005
NO_3_- (mg l^-1^)	0.052
NH_4_^+^ (mg l^-1^)	< 0.005

**Table 2.** Diatom indices where *Nitzschiatranstagensis* was found: IPS (Specific Pollution Index), Number of species, *H*’ (Shannon-Wiener diversity index), TDI (Trophic Diatom Index), CEE (European Index), IBD (Biological Diatom Index) and IDAP (Artois-Picardie Diatom Index).

**Index**	**Value**
IPS	18.7
Species richness	20.0
*H*′	2.3
TDI	11.2
CEE	12.2
IBD	17.3
IDAP	18.5
